# Microbial Communities Associated with Holothurians: Presence of Unique Bacteria in the Coelomic Fluid

**DOI:** 10.1264/jsme2.ME12020

**Published:** 2012-03-23

**Authors:** Masaki Enomoto, Satoshi Nakagawa, Tomoo Sawabe

**Affiliations:** 1 Laboratory of Microbiology, Faculty of Fisheries Sciences, Hokkaido University, 3–1–1 Minato-cho, Hakodate 041–8611, Japan

**Keywords:** holothurians, microbial diversity, coelomic fluid, host-microbe association, fisheries implications

## Abstract

Marine invertebrates interact with various microorganisms ranging from pathogens to symbionts. One-to-one symbiosis between a single microbial species and a single host animal has served as a model for the study of host-microbe interactions. In addition, increasing attention has recently been focused on the complex symbiotic associations, *e.g.*, associations between sponges and their symbionts, due to their biotechnological potential; however, relatively little is known about the microbial diversity associated with members of the phylum *Echinodermata*. Here, for the first time, we investigated microbial communities associated with a commercially important holothurian species, *Apostichopus japonicus*, using culture-dependent and -independent methods. Diverse and abundant heterotrophs, mostly *Gammaproteobacteria* members, were cultured semi-quantitatively. Using the cloning and sequencing technique, different microbial communities were found in different holothurian tissues. In the holothurian coelomic fluid, potentially metabolically active and phylogenetically unique members of *Epsilonproteobacteria* and *Rickettsiales* were discovered. This study suggests that coelomic fluids of marine invertebrates, at least those inhabiting intertidal areas where physical and chemical conditions fluctuate, provide microbes with unique and stable habitats.

Marine invertebrates harbor a wide array of ecto- and endo-symbiotic microbes. One-to-one symbiosis between a single microbial species and a single host animal has served as a model for the study of host-microbe interactions (7, 22, 37, 38, 39). Recently, increasing attention has been focused on complex symbiotic associations between a single host species and multiple microbial species/populations, *e.g.*, sponge symbiosis due to their biotechnological potential (57, 58). Many marine invertebrates, particularly sessile to nearly sessile, are rich sources of valuable metabolites (9, 14). An individual sponge harbors dozens of different microbial species, in which bacteria comprise up to 40% of total sponge volume (59). Sponge-dwelling bacteria or the sponge itself produce a variety of bioactive compounds showing antiviral, antitumor, and antimalarial activities (45, 58). From most phyla of marine invertebrates, host-associated microbial communities have been characterized; however, relatively little is known about microbial diversity within and on *Echinodermata* members such as sea stars, sea urchins, and sea cucumbers (19).

Holothurians are a group of marine invertebrates belonging to *Echinodermata*, and are distributed widely throughout the world from intertidal zones to the deep sea. Some holothurian species have a high market value and thus are heavily overexploited (18). Holothurians are model organisms for studying processes related to organ regeneration and organogenesis since they regenerate their digestive tract within a few weeks after self-evisceration (12, 42). Their body wall and coelomic fluid contain bioactive compounds with antibacterial, antifungal, anticoagulant, and antitumor activities (8, 17, 29). Although some microbes, *e.g.*, members of the genera *Pseudoalteromonas* and *Salegentibacter*, have been isolated in pure cultures from holothurians (1, 23, 34, 48), little is known about the associated microbial diversity.

In this study, we investigated microbial communities associated with a commercially important holothurian, *Apostichopus japonicus* (*Echinodermata: Holothuroidea*). We quantified culturable microorganisms in major microbial habitats in the holothurian body, including the body surface, digestive tract, and coelomic fluid. In addition, microbial diversity and niche segregation were comprehensively and quantitatively evaluated using culture-independent approaches.

## Materials and Methods

### Sampling and processing

Individuals of natural holothurians, *A. japonicus* (*Echinodermata: Holothuroidea*), were collected from the Funka Bay and Ainuma fishing port (42°08′N, 140°06′E), Hokkaido, Japan. Individuals (15.0–22.1 cm in length, 195–315.5 g) were rinsed with sterile 75% ASW (NaCl, 22.5 g; KCl, 0.5 g; MgSO_4_·7H_2_O, 4.0 g; MgCl_2_·6H_2_O, 8.1 g; CaSO_4_·2H_2_O, 1.0 g per liter) and were aseptically dissected into the small intestine, large intestine, body surface, and coelomic fluid. Briefly, outer surfaces of the body wall (12 cm^2^ per individual) were sampled using sterile cotton-tipped swabs (Men-tip; J.C.B, Industry, Tokyo, Japan). After wiping the body surface with 70% ethanol, the body wall was cut with a disposable scalpel blade to collect the coelomic fluid using a sterile syringe. Finally, small and large intestines were sampled. The cotton-tipped swabs and digestive tracts were homogenized in sterile 75% artificial seawater (ASW) using a stomacher (Pro-media, SH-IIM; ELMEX, Tokyo, Japan) (49). Samples were serially diluted in sterile 75% ASW for the cultivation test.

### Cell counting and cultivation test

Total cell counts were obtained by direct cell counting of the glutaraldehyde-fixed cells with DAPI (4′,6-diamidino-2-phenylindole) using epifluorescence microscopy (47). The abundance and diversity of culturable heterotrophs were estimated as previously described (56). After 7 days’ cultivation under 20°C, about thirty colonies were randomly picked up from plates. Isolates were physiologically characterized as previously described (49). In addition, 16S rRNA gene sequences of isolates were determined using the primers 24F and 1509R (Table 1). The PCR conditions were as follows: 30 cycles of 94°C for 1 min, 55°C for 1 min, and 72°C for 1.5 min. GoTaq Green Master mix (Promega, Madison, WI, USA) was used for the PCR reactions.

### 16S rRNA gene clone library

Microbial diversity was assessed for holothurians collected from the Ainuma fishing port. Microbial DNA was directly extracted from the microbial community in each homogenate sample or coelomic fluid with a Wizard Genomic DNA Purification Kit (Promega). DNA extracted from seawater surrounding the holothurian community was used as a positive control. To reduce PCR bias for the assessment of bacterial diversity, two primer sets, 24F-1509R and 24F-1540R, were used (Table 1). The PCR conditions were as follows: 35 cycles of 94°C for 1 min, 50°C for 1 min, and 72°C for 1.5 min. Each amplicon was excised and purified (Wizard SV Gel and PCR Clean-up System; Promega), and then ligated into the TOPO TA cloning vector (Invitrogen, Carlsbad, CA, USA) according to the instructions. Ligation products were transformed into *Escherichia coli* One Shot TOP10 cells (Invitrogen). Clones were amplified by PCR with vector-specific primers. The 920R primer was used for partial sequencing of the insert to determine the phylogenetic clone type (phylotype). Clones with ≥94% similarity were assigned to the same phylotype. Approximately 800 bp of each representative rRNA gene clone sequence was determined for both strands. To estimate the representation of the phylotypes, coverage was calculated by Good’s equation (13) with the formula, (1−[*n_1_/N*])×100, where *n_1_* is the number of single-occurrence phylotypes within a library and *N* is the number of clones examined. The bacterial community structures were compared by cluster analysis based on the clonal frequency of each representative phylotype. The square distance was determined by the Ward method (41).

### Construction of phylogenetic tree

The 16S rRNA gene sequences of representative clones and isolates were aligned with ARB software (25). Alignments were manually verified with known secondary structure regions. Phylogenetic analyses were restricted to nucleotide positions that could be unambiguously aligned. Phylogenetic trees were generated by a distance method using PAUP* 4.0b (54) and ARB. Distances were estimated with the Jukes-Cantor correction. Bootstrap analyses with 100 trial replications were used to obtain confidence estimates for the tree topologies.

### FISH analysis

Fluorescent *in-situ* hybridization was performed as described elsewhere (3, 21, 40, 51). In brief, cells were hybridized with the Cy3-labeled probes (Table 2) for 4.5 h at 46°C. The percentage of fluorescently-labeled cells to DAPI-stained cells was determined using epifluorescence microscopy.

### Nucleotide sequence accession numbers

The 16S rRNA gene sequences of the representative isolates and clones obtained in this study have been deposited in DDBJ/EMBL/GenBank under Accession No. AB550432 to AB550557.

## Results and Discussion

### Total cell counts and cultivation test

Total cell densities were estimated for the holothurian body surface, large and small intestines, and coelomic fluid from two individuals (Table 3). Unexpectedly, many prokaryotic cells were observed even in the coelomic fluid, which varied in the range of 4.8×10^5^ to 9.9×10^5^ cells mL^−1^. Cells observed in the coelomic fluid were mostly twisted rods (Fig. S1). The culturable heterotrophic populations generally accounted for less than 5% of the total cell counts (Table 3). In the sample Li-2 (the large intestine from the Ainuma individual), the population of culturable heterotrophs was probably overestimated, potentially due to the uneven distribution of cells in the large intestine homogenate.

### Culturable heterotrophs

**Fig. 1:**
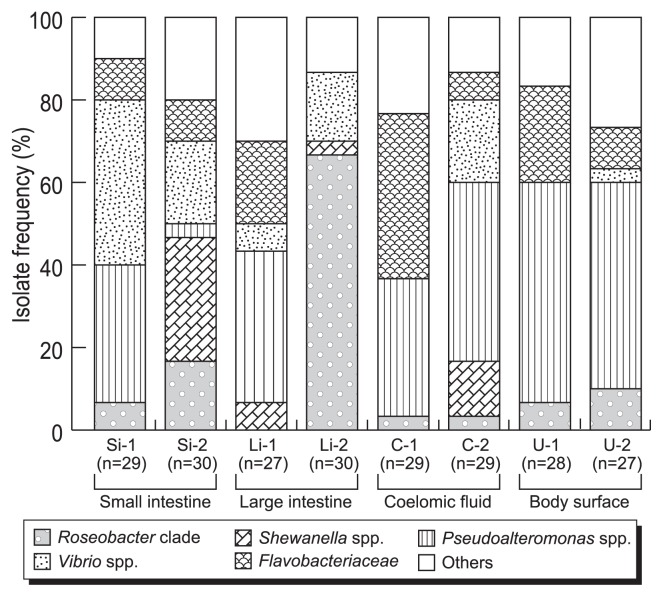
Composition of the culturable heterotrophs associated with holothurians. See Table 3 for sample codes.

There were marked differences in the composition of culturable microbial community in each sample (Fig. 1). Heterotrophic microorganisms associated with holothurians were represented by members of eight different phylogenetic groups within the phyla *Proteobacteria* and *Bacteroidetes: Roseobacter* clade, *Shewanella* spp., *Pseudoalteromonas* spp., *Vibrio* spp., and family *Flavobacteriaceae*. The most frequently recovered population consisted of members of the genus *Pseudoalteromonas* (Fig. 1 and Table S1), which accounted for up to 52% of total culturable heterotrophs. The physiological tests of culturable populations indicated that bacteria with the ability to utilize alginate are more frequently recovered from digestive tracts than from other body parts (Table S1). Abundant and diverse heterotrophs were recovered from holothurian coelomic fluids. In the coelomic fluid of Funka Bay individual (C-1), *Flavobacteriaceae* members comprised 27.6% of isolates. In contrast, *Vibrio* members represented the second most frequently recovered population in the Ainuma coelomic fluid (C-2). Considering the total cell counts and the composition of culturable heterotrophs, microbial communities found in the coelomic fluids cannot be regarded as contaminants from other body parts.

### Microbial communities evaluated by 16S rRNA gene library

**Fig. 2:**
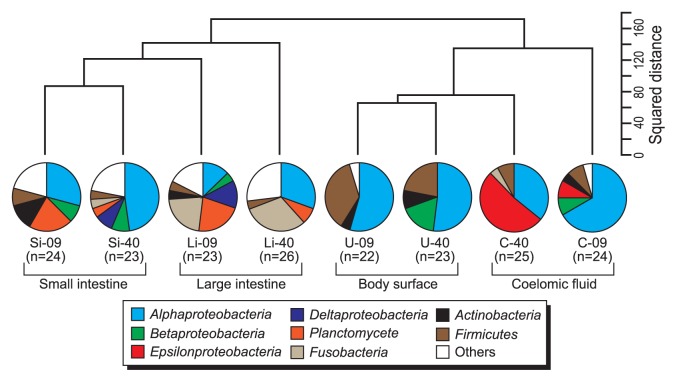
Similarity and composition of the bacterial population in holothurian tissues. The square distance (genetic similarity) was determined from the clonal frequency of each representative phylotype by the Ward method. Pie charts indicate the composition of bacterial population based on taxonomic grouping of 16S rRNA gene clone sequencing. See Table 3 for sample codes. 09: 24F-1509R primer set, 40: 24F-1540R primer set.

Bacterial 16S rRNA gene clone libraries were successfully constructed using two universal primer sets from all holothurian samples (Table 1). The archaeal 16S rRNA gene was not amplified from any samples used in this study, although archaeal diversity was previously assessed for the midgut contents of a deep-sea holothurian species (27). A total of 90 different bacterial phylotypes were identified from the 8 libraries on the basis of classification with ≥94% identity (Table S2). The coverage values were 58.3 and 70.0% (small intestine), 61.5 and 65.2% (large intestine), 86.4 and 95.7% (coelomic fluid), and 61.5 and 86.4% (body surface). A distinctive bacterial community was detected in each holothurian niche (Fig. 2).

**Fig. 3:**
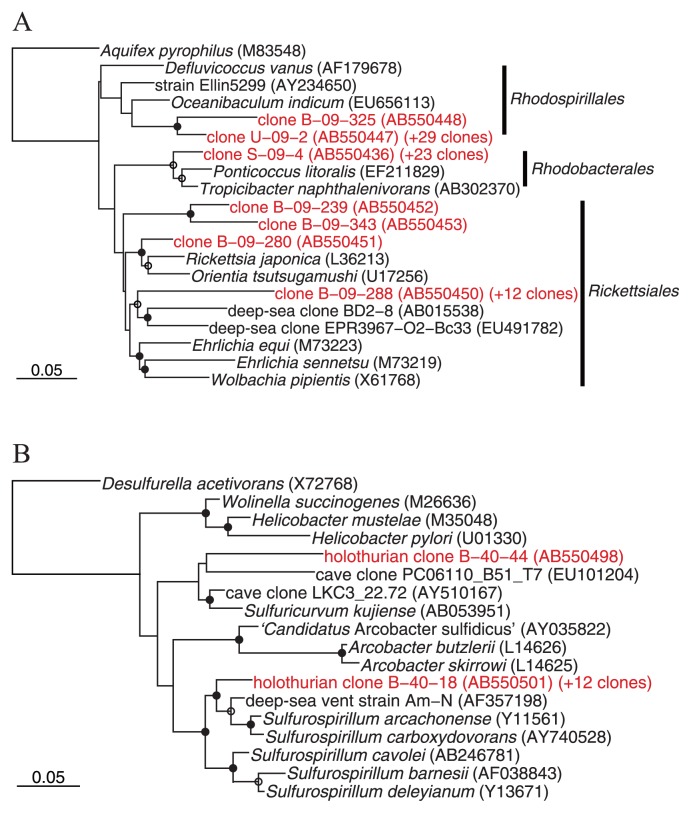
Phylogenetic tree including representative holothurian clones as determined by neighbor-joining analysis. (A) and (B) were respectively constructed from 424 and 528 sites of the rRNA gene sequence that could be unambiguously aligned. Clones sequenced in this study are shown in red. The clonal frequency of each representative clone obtained in this study and DDBJ accession numbers are shown in parentheses. Branch points conserved with bootstrap value of >75% (solid circles) and with bootstrap values of 50 to 74% (open circles) are indicated. Some groups are represented by shaded trapezoids that indicate the numbers of sequences. Scale bars represent 0.05 substitutions per nucleotide position. (A) Tree indicating the phylogenetic relationship among members of the *Alphaproteobacteria*. (B) Tree indicating the phylogenetic relationship among members of the *Epsilonproteobacteria*.

The rRNA gene clones affiliated to the class *Alphaproteobacteria* were dominantly detected in all holothurian samples (13.0–54.5% in clonal frequencies). Alphaproteobacterial clones mainly belonged to three different subgroups (Fig. 3A). *Roseobacter* clade was mainly detected in small and large intestines (up to 43.5% in clonal frequency). These clones were closely related to clones or isolates previously retrieved from various marine environments including coastal and pelagic seawater, sediments, and algae- and invertebrates-associated habitats (4, 5) (Table S2). Members of the genus *Defluviicoccus* were mainly detected from the body surface and the coelomic fluid (up to 54.5% in clonal frequency) (Table S2). Many alphaproteobacterial sequences from the coelomic fluid (28 and 37.5% in clonal frequencies; approximately half of the alphaproteobacterial clones) formed a novel cluster within the order *Rickettsiales* (Fig. 3A). Most *Rickettsiales* members have been recognized as obligate intracellular parasites of arthropods with the ability to infect vertebrates (44, 60). Recently, *Rickettsiales* have also been found in the cells of leeches and marine ciliates (20, 60, 62). This study confirmed that *Rickettsiales* members could associate with more diverse animals than previously recognized. Clones affiliated to *Planctomycetes* and *Fusobacteria* were dominantly detected from holothurian digestive tracts (Fig. 2). These bacteria are frequently retrieved from marine snow and sediments (15), and thus may be transported with sediment particles into holothurian digestive tracts. Clones of the class *Epsilonproteobacteria* were detected only from the coelomic fluid (8.3 and 52.0% in clonal frequencies) (Fig. 2 and Table S2). As in the case of *Rickettsiales* clones, epsilonproteobacterial clones formed a novel clade which distantly related to members of the genus *Sulfurospirillum* (up to 92% sequence similarity) (Fig. 3B). Members of the genus *Sulfurospirillum* are microaerobic sulfur reducers, which include two validly described marine species, *i.e.*, *S. carboxydovorans* and *S. arcachonense*(11, 16). Known marine habitats from which *Sulfurospirillum* relatives were frequently recovered are restricted to sulfidic environments such as deep-sea vents and whale falls (32). Although the symbiotic ability of the *Sulfurospirillum* members is unclear, deep-sea vent *Epsilonproteobacteria* members have the ability to associate with various invertebrates (30, 31, 33, 61).

### Whole-cell FISH analysis

**Fig. 4:**
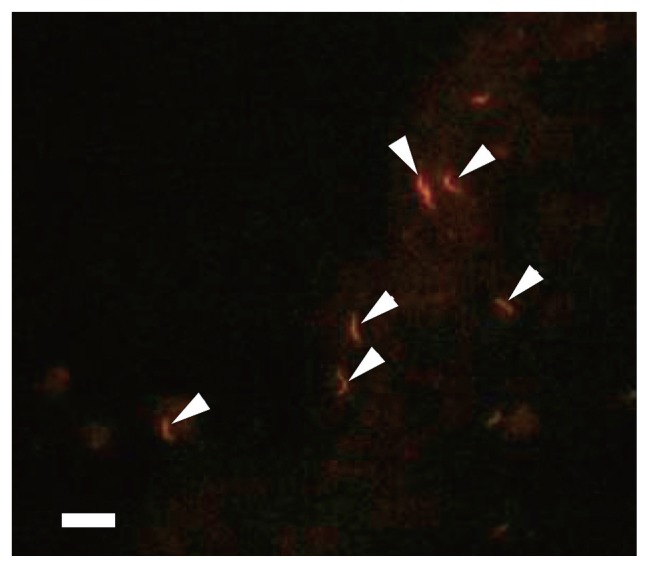
Epifluorescence micrograph of cells binding the *Epsilonpro-teobacteria*-specific probe (EP 402–423) in the holothurian coelomic fluid. Arrowheads indicate epsilonproteobacterial cells. Bar, 5 μm.

Microorganisms with high fluorescence emission after FISH hybridization have high growth rates and high metabolic activity (3). Metabolically active microbial populations were quantified using eight different fluorescent probes (Table 2). In general, FISH analysis confirmed the clone library data. *Bacteria* detected by the EUB338 probe accounted for approximately three fourths of total cells in all holothurian samples (Table 4). *Epsilonproteobacteria* specific probe-binding cells were observed only in the coelomic fluid (Fig. 4). ALF968 probe-binding cells were abundantly found in all holothurian samples. These cells existed freely even in the coelomic fluid where *Rickettsiales* members accounted for half of the alphaproteobacterial clones, suggesting that *Rickettsiales* members detected in the coelomic fluid lack the ability to inhabit holothurian cells.

## Conclusion

This study revealed for the first time the diverse and abundant microbial community associated with the holothurian body. In particular, it was remarkable that probably metabolically active and phylogenetically unique *Epsilonproteobacteria* and *Rickettsiales* members were discovered in the holothurian coelomic fluid, which contains antibacterial compounds (8). In addition, *Echinodermata* immunocytes produce reactive oxygen species (ROS) when in contact with bacteria or bacterial cell wall proteins (28). Nevertheless, potentially pathogenic microbes and fungi were repeatedly isolated from the holothurian coelomic fluid (1, 46). In addition, the coelomic fluids of oysters and abalones were used as inocula for isolating novel microorganisms (10, 24, 52). Echinoderms, at least those inhabiting intertidal areas where physical and chemical conditions fluctuate, have increased ability to maintain homeostasis in coelomic fluid (6). These suggest that coelomic fluid of marine invertebrates provides various oceanic microorganisms with unique, stable habitats. Focal points raised by this study for future research include: time course and evolution of symbiont diversity, microbial metabolism, host-microbe interactions and potential biotechnological and fisheries implications.

## Supplementary Material



## Figures and Tables

**Table 1:** Primers used in this study

Primer	Target	Sequence (5′-3′)	Position (*E. coli*)	Reference
24F	*Bacteria*	AGAGTTTGATCCTGGCT	8 to 24	(50)
1509R	*Bacteria*	GGCTACCTTGTTACGACT	1,510 to 1,493	(50)
1540R	*Bacteria*	AAGGAGGTGATCCAGCCGCA	1,541 to 1,522	(50)
Arch21F	*Archaea*	TTCCGGTTGATCCYGCCGGA	7 to 26	(3)
Arch915R	*Archaea*	GTGCTCCCCCGCCAATTCCT	935 to 915	(3)
920R	*Bacteria*	CCCCGTCAATTCCTTTGAGT	928 to 909	(50)

**Table 2:** Oligonucleotide probes and competitors used in this study

Probe or competitor	Specificity	Sequence (5′-3′)	Target RNA	Position (*E. coli*)	FA (%)	Reference
Probe ARCH915	*Archaea*	GTGCTCCCCCGCCAATTCCT	16S	915–935	20	(53)
Probe EUB338	*Bacteria*	GCTGCCTCCCGTAGGAGT	16S	338–355	20	(2)
Probe ALF968	*Alphaproteobacteria*	GGTAAGGTTCTGCGCGTT	16S	968–986	20	(35)
Probe GAM42a	*Gammaproteobacteria*	GCCTTCCCACATCGTTT	23S	1,027–1,043	35	(26)
Probe BET42a	*Betaproteobacteria*	GCCTTCCCACTTCGTTT	23S	1,027–1,043	35	(26)
Probe EP402-423	*Epsilonproteobacteria*	GAAAKGYGTCATCCTCCACG	16S	402–423	30	(55)
Probe CFB560	*Bacteroidetes*	WCCCTTTAAACCCART	16S	560–575	30	(43)
Probe PLA886	*Planctomycetes*	GCCTTGCGACCATACTCCC	16S	886–904	35	(36)
Competitor GAM42a_C	*Gammaproteobacteria*	GCCTTCCCACTTCGTTT	23S	1,027–1,043		(26)
Competitor BET42a_C	*Betaproteobacteria*	GCCTTCCCACATCGTTT	23S	1,027–1,043		(26)

**Table 3:** Total cell counts and culturable population from the holothurian tissues

Sample code^a^	Total cell counts^b^	Culturable cell counts^c^	Percentage of culturable cells (%)
Si-1	4.1×10^6^	7.6×10^4^	1.9
Si-2	6.1×10^5^	1.8×10^4^	3.0
Li-1	3.6×10^6^	8.8×10^4^	2.4
Li-2	3.3×10^5^	2.6×10^5^	78.8
U-1	1.2×10^6^	1.1×10^4^	0.9
U-2	3.8×10^5^	1.7×10^3^	0.5
C-1	9.9×10^5^	8.4×10^3^	0.9
C-2	4.8×10^5^	1.3×10^3^	0.3

a Codes denote holothurian body parts and geographical origin. Si, Small intestine; Li, Large intestine; U, Surface; C, Coelomic fluid; 1, Funka Bay; 2, Ainuma Fishing Port.

b Si-1, Si-2, Li-1, and Li-2, cells g^−1^; U-1 and U-2, cells cm^−2^; C-1 and C-2, cells mL^−1^.

c Si-1, Si-2, Li-1, and Li-2, CFU g^−1^; U-1 and U-2, CFU cm^−2^; C-1 and C-2, CFU mL^−1^.

**Table 4:** FISH-direct count analysis of metabolically active microbial populations associated with holothurians

Samples	Total cell counts±SD (10^6^ cells unit^−1^)	% of DAPI stained cells±SD							

		EUB338	ARCH915	ALF968	BET42a	GAM42a	EP402-423	CFB560	PLA886
Small intestine	22.1±3.0	72.6±9.2	ND	27.8±3.9	7.0±0.8	2.5±0.4	ND	4.0±1.2	12.2±0.2
Large intestine	23.9±0.9	74.4±9.8	ND	13.9±2.1	3.6±2.2	3.4±1.4	ND	3.0±0.3	7.8±1.7
Surface	2.2±0.3	77.9±6.5	ND	46.9±7.5	3.9±3.5	9.6±2.3	ND	ND	ND
Coelomic fluid	0.4±0.1	76.3±4.4	ND	45.2±2.2	ND	2.5±0.3	21.3±3.1	ND	ND

SD, standard deviation (*n*=4).

ND, not detected.

## References

[1] Alekseeva SA, Bakunina IY, Nedashkovskaya OI, Isakov VV, Mikhailov VV, Zvyagintseva TN (2004). Intracellular alginolytic enzymes of the marine bacterium *Pseudoalteromonas citrea* KMM 3297. Biochemistry (Moscow).

[2] Amann RI, Binder BJ, Olson RJ, Chisholm SW, Devereux R, Stahl DA (1990). Combination of 16S rRNA-targeted oligonucleotide probes with flow cytometry for analyzing mixed microbial populations. Appl Environ Microbiol.

[3] Amann RI, Ludwig W, Schleifer KH (1995). Phylogenetic identification and in situ detection of individual microbial cells without cultivation. Microbiol Rev.

[4] Brinkhoff T, Giebel HA, Simon M (2008). Diversity, ecology, and genomics of the Roseobacter clade: a short overview. Arch Microbiol.

[5] Buchan A, González JM, Moran MA (2005). Overview of the marine roseobacter lineage. Appl Environ Microbiol.

[6] Davenport J (1985). Osmotic control in marine animals. Symp Soc Exp Biol.

[7] Dubilier N, Bergin C, Lott C (2008). Symbiotic diversity in marine animals: the art of harnessing chemosynthesis. Nat Rev Microbiol.

[8] Dybas L, Fankboner PV (1986). Holothurian survival strategies: mechanisms for the maintenance of a bacteriostatic environment in the coelomic cavity of the sea cucumber, *Parastichopus californicus*. Dev Comp Immunol.

[9] Egan S, Thomas T, Kjelleberg S (2008). Unlocking the diversity and biotechnological potential of marine surface associated microbial communities. Curr Opin Microbiol.

[10] Faury N, Saulnier D, Thompson FL, Gay M, Swings J, Le Roux F (2004). *Vibrio crassostreae* sp. nov., isolated from the haemolymph of oysters (*Crassostrea gigas*). Int J Syst Evol Microbiol.

[11] Finster K, Liesack W, Tindall BJ (1997). *Sulfurospirillum arcachonense* sp. nov., a new microaerophilic sulfur-reducing bacterium. Int J Syst Bacteriol.

[12] García-Arrarás JE, Greenberg MJ (2001). Visceral regeneration in holothurians. Microsc Res Tech.

[13] Good IJ (1953). The population frequencies of species and the estimation of population parameters. Biometrika.

[14] Haygood MG, Schmidt EW, Davidson SK, Faulkner DJ (1999). Microbial symbionts of marine invertebrates: opportunities for microbial biotechnology. J Mol Microbiol Biotechnol.

[15] Hieu CX, Voigt B, Albrecht D, Becher D, Lombardot T, Glöckner FO, Amann R, Hecker M, Schweder T (2008). Detailed proteome analysis of growing cells of the planctomycete *Rhodopirellula baltica* SH1^T^. Proteomics.

[16] Jensen A, Finster K (2005). Isolation and characterization of *Sulfurospirillum carboxydovorans* sp. nov., a new microaerophilic carbon monoxide oxidizing epsilon Proteobacterium. Antonie Van Leeuwenhoek.

[17] Jin JO, Shastina VV, Shin SW (2009). Differential effects of triterpene glycosides, frondoside A and cucumarioside A2-2 isolated from sea cucumbers on caspase activation and apoptosis of human leukemia cells. FEBS Lett.

[18] Kelly MS (2005). Echinoderms: their culture and bioactive compounds. Prog Mol Subcell Biol.

[19] Kelly MS, McKenzie JD (1995). Survey of the occurrence and morphology of sub-cuticular bacteria in shelf echinoderms from the north-east Atlantic Ocean. Mar Biol.

[20] Kikuchi Y, Sameshima S, Kitade O, Kojima J, Fukatsu T (2002). Novel clade of *Rickettsia* spp. from leeches. Appl Environ Microbiol.

[21] Kindaichi T, Awata T, Suzuki Y, Tanabe K, Hatamoto M, Ozaki N, Ohashi A (2011). Enrichment using an up-flow column reactor and community structure of marine anammox bacteria from coastal sediment. Microbes Environ.

[22] Kubota N, Kanemori M, Sasayama Y, Aida M, Fukumori Y (2007). Identification of endosymbionts in *Oligobrachia mashikoi*(Siboglinidae, Annelida). Microbes Environ.

[23] Kurahashi M, Fukunaga Y, Sakiyama Y, Harayama S, Yokota A (2009). *Iamia majanohamensis* gen. nov., sp. nov., an actinobacterium isolated from sea cucumber *Holothuria edulis*, and proposal of *Iamiaceae* fam. nov. Int J Syst Evol Microbiol.

[24] Le Roux F, Goubet A, Thompson FL, Faury N, Gay M, Swings J, Saulnier D (2005). *Vibrio gigantis* sp. nov., isolated from the haemolymph of cultured oysters (*Crassostrea gigas*). Int J Syst Evol Microbiol.

[25] Ludwig W, Strunk O, Westram R (2004). ARB: a software environment for sequence data. Nucleic Acids Res.

[26] Manz W, Amann R, Ludwig W, Wagner M, Schleifer KH (1992). Phylogenetic oligodeoxynucleotide probes for the major subclasses of proteobacteria: problems and solutions. Syst Appl Microbiol.

[27] McInerney JO, Wilkinson M, Patching JW, Embley TM, Powell R (1995). Recovery and phylogenetic analysis of novel archaeal rRNA sequences from a deep-sea deposit feeder. Appl Environ Microbiol.

[28] Mydlarz LD, Jones LE, Harvell CD (2006). Innate immunity, environmental drivers, and disease ecology of marine and freshwater invertebrates. Annu Rev Ecol Evol Syst.

[29] Nagase H, Enjyoji K, Shima M, Kitazato K, Yoshioka A, Saito H, Kato H (1996). Effect of depolymerized holothurian glycosaminoglycan (DHG) on the activation of factor VIII and factor V by thrombin. J Biochem.

[30] Nakagawa S, Takai K, Inagaki F (2005). Variability in microbial community and venting chemistry in a sediment-hosted backarc hydrothermal system: Impacts of subseafloor phase-separation. FEMS Microbiol Ecol.

[31] Nakagawa S, Takai K, Inagaki F, Hirayama H, Nunoura T, Horikoshi K, Sako Y (2005). Distribution, phylogenetic diversity and physiological characteristics of epsilon-*Proteobacteria* in a deep-sea hydrothermal field. Environ Microbiol.

[32] Nakagawa S, Takaki Y (2009). Nonpathogenic Epsilonproteo-bacteria. Encyclopedia of Life Sciences.

[33] Nakagawa S, Takaki Y, Shimamura S, Reysenbach A.-L, Takai K, Horikoshi K (2007). Deep-sea vent epsilon-proteobacterial genomes provide insights into emergence of pathogens. Proc Natl Acad Sci USA.

[34] Nedashkovskaya OI, Suzuki M, Vancanneyt M, Cleenwerck I, Zhukova NV, Vysotskii MV, Mikhailov VV, Swings J (2004). *Salegentibacter holothuriorum* sp. nov., isolated from the edible holothurian *Apostichopus japonicus*. Int J Syst Evol Microbiol.

[35] Neef A (1997). Anwendung der *in situ*-Einzelzell-identifizierung von bakterien zur populationsanalyse in komplexen mikrobiellen Biözonosen. Ph.D. thesis.

[36] Neef A, Amann R, Schlesner H, Schleifer KH (1998). Monitoring a widespread bacterial group: in situ detection of planctomycetes with 16S rRNA-targeted probes. Microbiology.

[37] Nussbaumer AD, Fisher CR, Bright M (2006). Horizontal endosymbiont transmission in hydrothermal vent tubeworms. Nature.

[38] Nyholm SV, McFall-Ngai MJ (2004). The winnowing: establishing the squid-vibrio symbiosis. Nat Rev Microbiol.

[39] Okabe S, Oshiki M, Kamagata Y (2010). A great leap forward in microbial ecology. Microbes Environ.

[40] Ootsubo M, Shimizu T, Tanaka R, Sawabe T, Tajima K, Yoshimizu M, Ezura Y, Ezaki T, Oyaizu H (2002). Oligonucleotide probe for detecting Enterobacteriaceae by in situ hybridization. J Appl Microbiol.

[41] Orloci L (1967). An agglomerate method for classification of plant communities. J Ecol.

[42] Ortiz-Pineda PA, Ramírez-Gómez F, Pérez-Ortiz J (2009). Gene expression profiling of intestinal regeneration in the sea cucumber. BMC Genomics.

[43] O’Sullivan LA, Weightman AJ, Fry JC (2002). New degenerate *Cytophaga-Flexibacter-Bacteroides*-specific 16S ribosomal DNA-targeted oligonucleotide probes reveal high bacterial diversity in River Taff epilithon. Appl Environ Microbiol.

[44] Perlman SJ, Hunter MS, Zchori-Fein E (2006). The emerging diversity of *Rickettsia*. Proc Biol Sci.

[45] Piel J, Butzke D, Fusetani N, Hui D, Platzer M, Wen G, Matsunaga S (2005). Exploring the chemistry of uncultivated bacterial symbionts: antitumor polyketides of the pederin family. J Nat Prod.

[46] Pivkin MV (2000). Filamentous fungi associated with holothurians from the sea of Japan, off the primorye coast of Russia. Biol Bull.

[47] Porter KG, Feig YS (1980). The use of DAPI for identifying and counting aquatic microflora. Limnol Oceanogr.

[48] Sakai T, Ishizuka K, Kato I (2003). Isolation and characterization of a fucoidan-degrading marine bacterium. Mar. Biotechnol (NY).

[49] Sawabe T, Oda Y, Ezura Y (1995). Alginate degradation by bacteria isolated from the gut of sea urchins and abalones. Microb Ecol.

[50] Sawabe T, Sugimura I, Ohtsuka M, Nakano K, Tajima K, Ezura Y, Christen R (1998). *Vibrio halioticoli* sp. nov., a non-motile alginolytic marine bacterium isolated from the gut of the abalone *Haliotis discus hannai*. Int J Syst Bacteriol.

[51] Sawabe T, Yoshizawa A, Kawanishi Y (2009). Multi-probe-fluorescence in situ hybridization for the rapid enumeration of viable *Vibrio parahaemolyticus*. Microbes Environ.

[52] Schlösser A, Lipski A, Schmalfuss J, Kugler F, Beckmann G (2008). *Oceaniserpentilla haliotis* gen. nov., sp. nov., a marine bacterium isolated from haemolymph serum of blacklip abalone. Int J Syst Evol Microbiol.

[53] Stahl DA, Amann RI, Stackebrandt E, Goodfellow M (1991). Development and application of nucleic acid probes. Nucleic Acid Techniques in Bacterial Systematics.

[54] Swofford DL (2000). PAUP* Phylogenetic analysis using parsimony (and other methods), version 4.

[55] Takai K, Oida H, Suzuki Y, Hirayama H, Nakagawa S, Nunoura T, Inagaki F, Nealson KH, Horikoshi K (2004). Spatial distribution of marine Crenarchaeota Group I in the vicinity of deep-sea hydrothermal systems. Appl Environ Microbiol.

[56] Tanaka R, Sugimura I, Sawabe T, Yoshimizu M, Ezura Y (2003). Gut microflora of abalone *Haliotis discus* hannai in culture changes coincident with a change in diet. Fish Sci.

[57] Taylor MW, Hill RT, Piel J, Thacker RW, Hentschel U (2007). Soaking it up: the complex lives of marine sponges and their microbial associates. ISME J.

[58] Taylor MW, Radax R, Steger D, Wagner M (2007). Sponge-associated microorganisms: evolution, ecology, and biotechnological potential. Microbiol Mol Biol Rev.

[59] Vacelet J (1975). Etude en microscopie electronique de l’association entre bacteries et spongiaires du genre Verongia (Dictyoceratida). J. Microsc. Biol Cell.

[60] Vannini C, Petroni G, Verni F, Rosati G (2005). A bacterium belonging to the *Rickettsiaceae* family inhabits the cytoplasm of the marine ciliate *Diophrys appendiculata*(Ciliophora, Hypotrichia). Microb Ecol.

[61] Watsuji T, Nakagawa S, Tsuchida S, Toki T, Hirota A, Tsunogai U, Takai K (2010). Diversity and function of epibiotic microbial communities on the galatheid crab, *Shinkaia crosnieri*. Microbes Environ.

[62] Weinert LA, Werren JH, Aebi A, Stone GN, Jiggins FM (2009). Evolution and diversity of *Rickettsia* bacteria. BMC Biol.

