# Supplementary material for: Microbial Communities Associated with Holothurians: Presence of Unique Bacteria in the Coelomic Fluid
Source: Microbes Environ. 2012 Mar 23;27(3):300–5. doi: 10.1264/jsme2.ME12020 (PMC4036045; doi:10.1264/jsme2.ME12020)
Supplement: Supplementary file 1 [file 27_300_s1.pdf]

Table S1. Physiological and phylogenetic characteristics of strains isolated from the holothurian tissues. See Table 1 for sample codes.

Origin	Strain	Gram stain	Morphology	Motility	Flagellum	OF test	Na^+ requirement	Oxidase	Catalase	DNase	Gelatinase	Agarase	Luminescence	Spore formation	Caseinase	Amylase	Growth at 4 degC	Growth at 15 degC	Growth at 25 degC	Growth at 30 degC	Growth at 37 degC	Lipase (Tween20)	Lipase (Tween80)	Alginase	Fucoidanase	Chitinase	Elastinase	Growth on TCBS	Pigmentation	Taxa
Si-1	S-1-1	-	rod	-	-	O	M	+	+	+	-	-	-	-	+	+	-	+	+	+	-	+	+	-	-	-	+	-	-	*Pseudoalteromonas marina*
	S-1-2	-	rod	-	-	O	M	+	+	+	-	-	-	-	+	+	-	+	+	+	-	+	+	-	-	-	+	-	-	*Pseudoalteromonas marina*
	S-1-3	-	rod	-	-	O	M	+	+	-	+	-	-	-	-	-	-	+	+	+	-	+	-	-	-	-	-	-	-	*Marinomonas pontica*
	S-1-4	-	rod	+	polar	F	M	+	+	+	+	-	-	-	+	+	-	+	+	-	-	+	+	-	-	-	-	+	-	*Vibrio splendidus*
	S-1-5	-	rod	+	polar	F	M	+	+	+	-	-	-	-	+	+	-	+	+	-	-	+	+	-	-	-	-	+	-	*Vibrio splendidus*
	S-1-6	-	rod	-	-	O	M	+	+	+	-	-	-	-	+	+	-	+	+	+	-	+	+	-	-	-	-	-	-	*Pseudoalteromonas marina*
	S-1-7	-	rod	+	polar	F	M	+	+	+	-	-	-	-	-	+	-	+	+	-	-	+	+	+	-	+	-	+	-	*Vibrio splendidus*
	S-1-8	-	rod	+	polar	O	M	+	+	+	-	-	-	-	+	-	-	+	+	+	-	+	+	-	-	-	-	-	-	*Pseudoalteromonas elyakovii*
	S-1-9	-	rod	-	-	O	M	+	+	+	-	-	-	-	+	+	-	+	+	+	-	+	+	+	-	-	-	-	-	*Pseudoalteromonas marina*
	S-1-10	-	rod	+	polar	F	M	+	+	+	+	-	-	-	-	+	-	+	+	-	-	+	+	+	-	+	-	+	-	*Vibrio splendidus*
	S-1-11	-	rod	+	polar	O	M	+	+	+	+	+	-	-	+	-	-	+	+	+	-	+	+	+	-	-	-	-	-	*Pseudoalteromonas elyakovii*
	S-1-12	-	rod	-	-	O	M	+	+	+	-	+	-	-	+	-	-	+	+	+	-	+	+	+	-	-	-	-	-	*Pseudoalteromonas elyakovii*
	S-1-13	-	rod	-	-	O	M	+	+	+	-	-	-	-	-	-	-	+	+	-	-	+	+	-	-	-	-	-	orange	*Winogradskyella thalassocola*
	S-1-14	-	rod	-	-	O	M	+	+	+	-	-	-	+	+	+	-	+	+	+	-	+	+	-	-	-	-	-	-	*Pseudoalteromonas marina*
	S-1-15	-	rod	-	-	O	M	+	+	+	-	-	-	-	-	-	-	+	+	-	-	+	+	-	-	-	-	-	-	*Colwellia aestuarii*
	S-1-16	-	rod	+	polar	F	M	+	+	+	-	-	-	-	+	+	-	+	+	+	-	+	+	-	-	-	-	-	-	*Vibrio cyclitrophicus*
	S-1-18	-	rod	-	-	O	M	+	+	+	-	-	-	-	-	-	-	+	+	-	-	+	-	-	-	-	-	-	orange	*Lacinutrix* sp.
	S-1-19	-	rod	+	polar	F	M	+	+	+	+	-	-	-	+	+	-	+	+	-	-	+	+	+	-	+	-	+	-	*Vibrio splendidus*
	S-1-20	-	rod	-	-	O	M	+	+	+	-	-	-	-	-	-	-	+	+	-	-	+	-	-	-	-	-	-	orange	*Bacteroidetes* bacterium
	S-1-21	-	rod	-	-	O	M	+	+	+	-	-	-	-	+	+	-	+	+	+	-	+	+	-	-	-	-	-	-	*Pseudoalteromonas marina*
	S-1-22	-	rod	+	polar	F	M	+	+	+	+	-	-	-	-	+	-	+	+	+	-	+	+	-	-	-	-	-	-	*Vibrio cyclitrophicus*
	S-1-23	-	rod	+	polar	F	M	+	+	+	-	-	-	-	-	+	-	+	+	-	-	+	+	+	-	-	-	+	-	*Vibrio splendidus*
	S-1-24	-	rod	-	-	O	M	-	+	-	+	-	-	-	-	-	-	+	+	+	-	-	-	-	-	-	-	-	-	*Marinomonas pontica*
	S-1-25	-	rod	+	polar	F	M	+	+	+	-	-	-	-	-	-	-	+	+	+	-	+	+	+	-	+	-	+	-	*Vibrio splendidus*
	S-1-26	-	rod	+	polar	F	M	+	+	+	-	-	-	-	-	-	-	+	+	+	-	+	+	+	-	+	-	+	-	*Vibrio splendidus*
	S-1-27	-	rod	+	polar	F	M	+	+	+	-	-	-	-	+	-	-	+	+	-	-	+	+	+	-	+	-	+	-	*Vibrio splendidus*
	S-1-28	-	rod	-	-	O	M	+	+	+	-	-	-	-	-	-	-	+	+	-	-	+	-	-	-	-	-	-	-	*Rhodobacterales* bacterium
	S-1-29	-	rod	+	polar	F	M	+	+	+	+	-	-	-	+	-	-	+	+	-	-	+	+	+	-	+	-	+	-	*Vibrio splendidus*
	S-1-30	-	rod	+	polar	O	M	+	+	+	+	+	-	-	+	+	-	+	+	+	-	+	+	-	-	-	-	-	-	*Pseudoalteromonas arctica*

Table S1. Continued.

Origin	Strain	Gram stain	Morphology	Motility	Flagellum	OF test	Na^+ requirement	Oxidase	Catalase	DNase	Gelatinase	Agarase	Luminescence	Spore formation	Caseinase	Amylase	Growth at 4 degC	Growth at 15 degC	Growth at 25 degC	Growth at 30 degC	Growth at 37 degC	Lipase (Tween20)	Lipase (Tween80)	Alginase	Fucoidanase	Chitinase	Elastinase	Growth on TCBS	Pigmentation	Taxa
Si-2	S-2-1	-	rod	-	-	N	M	+	+	-	-	-	-	-	-	+	-	+	+	-	-	+	+	-	-	-	-	-	-	*Roseobacter* sp.
	S-2-2	-	rod	-	-	F	M	+	+	+	-	+	-	-	-	+	-	+	+	-	-	+	+	+	-	-	-	+	brown	*Shewanella baltica*
	S-2-3	-	rod	+	polar	F	M	+	+	+	-	-	-	-	+	-	-	+	+	-	-	+	+	+	-	+	-	+	-	*Vibrio splendidus*
	S-2-4	-	rod	-	-	O	M	-	+	+	-	-	-	-	-	-	-	+	+	+	-	+	+	-	-	-	-	+	-	*Pseudomonas* sp.
	S-2-5	-	rod	-	-	F	M	+	+	+	-	-	-	-	-	-	-	+	+	-	-	+	+	+	-	-	-	+	brown	*Shewanella baltica*
	S-2-6	-	rod	-	-	O	M	+	+	+	-	-	-	-	-	+	-	+	+	-	-	+	+	-	-	-	-	+	yellow	*Shewanella kaireitica*
	S-2-7	-	rod	+	polar	F	M	+	+	+	-	-	-	-	-	+	-	+	+	-	-	+	+	+	-	-	-	+	brown	*Shewanella baltica*
	S-2-8	-	rod	+	polar	O	M	+	+	-	-	-	-	-	-	-	-	+	+	+	-	+	+	-	-	-	+	-	cream	*Pseudoalteromonas prydzensis*
	S-2-9	-	rod	+	polar	F	M	+	+	+	-	+	-	-	+	-	-	+	+	-	-	+	+	+	-	-	-	+	-	*Shewanella baltica*
	S-2-10	-	rod	+	polar	F	M	+	+	+	-	-	-	-	+	+	-	+	+	-	-	+	+	-	-	+	-	+	-	*Vibrio tasmaniensis*
	S-2-11	-	rod	-	-	F	M	+	+	+	-	+	-	-	+	-	-	+	+	-	-	+	+	+	-	-	-	+	brown	*Shewanella pacifica*
	S-2-12	-	rod	-	-	F	M	+	+	+	+	+	-	-	+	-	-	+	+	-	-	+	+	+	-	-	-	+	brown	*Shewanella baltica*
	S-2-13	-	coccus	-	-	F	M	-	+	+	-	+	-	-	-	-	-	+	+	+	-	-	-	-	-	-	-	-	yellow	*Sphingobacteriales* bacterium
	S-2-14	-	rod	+	polar	F	M	+	+	-	-	-	-	-	-	+	-	+	-	-	-	+	+	-	-	+	-	-	cream	*Aliivibrio logei*
	S-2-15	-	rod	-	-	F	M	-	-	-	-	+	-	-	-	-	-	+	+	+	-	+	-	-	-	-	-	-	yellow	*Sphingobacteriales* bacterium
	S-2-16	-	rod	-	-	F	M	-	-	+	-	+	-	-	-	+	-	+	+	-	-	+	+	-	-	-	-	-	yellow	*Sphingobacteriales* bacterium
	S-2-17	-	rod	+	polar	F	M	+	+	-	-	-	-	+	-	+	-	+	-	-	-	+	+	+	-	-	-	+	-	*Vibrio splendidus*
	S-2-18	-	rod	+	polar	F	M	+	+	-	-	-	-	+	+	-	-	+	+	-	-	+	+	-	-	+	-	+	-	*Vibrio tasmaniensis*
	S-2-19	-	rod	-	-	F	M	+	+	+	-	-	-	-	-	-	-	+	+	+	-	+	+	+	-	-	-	+	-	*Shewanella baltica*
	S-2-20	-	rod	-	-	F	M	+	+	+	-	-	-	-	-	-	-	+	+	-	-	+	+	+	-	-	-	+	cream	*Shewanella baltica*
	S-2-21	-	coccus	-	-	O	M	-	+	-	-	-	-	-	-	-	-	+	-	-	-	+	+	-	-	-	-	-	yellow	*Colwelliaceae* bacterium
	S-2-22	-	rod	-	-	N	M	-	+	-	-	-	-	-	-	+	-	+	-	-	-	+	+	-	-	-	-	-	brown	*Colwellia* sp.
	S-2-23	-	rod	+	polar	F	M	+	+	+	-	-	-	-	+	-	-	+	+	-	-	+	+	-	-	+	-	+	-	*Vibrio tasmaniensis*
	S-2-24	+	rod	-	-	O	T	+	+	+	-	-	-	+	+	-	-	+	+	+	-	+	+	-	-	-	-	-	-	*Bacillus cereus*
	S-2-25	-	rod	+	polar	F	M	+	+	+	-	-	-	-	+	-	-	+	+	-	-	+	+	-	-	+	-	+	cream	*Vibrio tasmaniensis*
	S-2-26	-	rod	-	-	O	M	+	+	-	-	-	-	-	-	-	-	+	+	-	-	+	+	-	-	-	-	-	-	*Roseobacter* sp.
	S-2-27	-	rod	-	-	F	M	+	+	+	+	+	-	-	-	-	-	+	+	-	-	+	+	+	-	-	-	+	orange	*Shewanella baltica*
	S-2-28	-	rod	-	-	O	M	+	+	-	-	-	-	-	-	-	-	+	+	-	-	+	+	-	-	-	-	-	-	*Roseobacter* sp.
	S-2-29	-	rod	-	-	O	M	+	+	-	-	-	-	-	-	+	-	+	+	-	-	+	+	-	-	-	-	-	-	*Roseobacter* sp.
	S-2-30	-	rod	-	-	O	M	+	+	-	-	-	-	-	-	-	-	+	+	-	-	+	+	-	-	-	-	-	-	*Roseobacter* sp.

Table S1. Continued.

Origin	Strain	Gram stain	Morphology	Motility	Flagellum	OF test	Na^+ requirement	Oxidase	Catalase	DNase	Gelatinase	Agarase	Luminescence	Spore formation	Caseinase	Amylase	Growth at 4 degC	Growth at 15 degC	Growth at 25 degC	Growth at 30 degC	Growth at 37 degC	Lipase (Tween20)	Lipase (Tween80)	Alginase	Fucoidanase	Chitinase	Elastinase	Growth on TCBS	Pigmentation	Taxa
Li-1	L-1-1	-	rod	-	-	O	M	+	+	+	+	-	-	-	+	-	-	+	+	+	-	+	+	+	-	-	-	-	orange	*Psychroserpens burtonensis*
	L-1-2	+	coccus	-	-	O	M	-	+	+	+	-	-	-	+	-	-	+	+	+	-	+	+	-	-	-	-	+	yellow	*Micrococcus* sp.
	L-1-3	-	rod	+	polar	O	M	+	+	+	-	-	-	-	-	-	-	+	+	+	-	+	-	-	-	-	-	-	-	*Marinomonas pontica*
	L-1-4	-	rod	-	-	O	M	+	+	+	+	-	-	-	+	-	-	+	+	+	-	+	+	+	-	-	-	-	orange	*Lacinutrix* sp.
	L-1-5	-	rod	-	-	O	M	+	+	+	+	+	-	-	+	+	-	+	+	+	-	+	+	-	-	-	-	-	orange	*Zobellia russellii*
	L-1-6	-	rod	-	-	O	M	+	+	-	-	-	-	-	-	-	-	+	+	-	-	+	+	-	-	-	-	-	orange	*Bacteroidetes bacterium*
	L-1-8	-	rod	-	-	O	M	+	+	-	+	-	-	-	-	-	-	+	+	-	-	+	+	-	-	-	-	-	orange	*Lacinutrix copepodicola*
	L-1-9	-	rod	-	-	O	M	+	+	+	+	-	-	-	+	+	-	+	+	+	-	+	+	-	-	-	-	-	-	*Pseudoalteromonas marina*
	L-1-10	-	rod	-	-	O	M	+	+	-	-	-	-	-	-	-	-	+	+	+	-	-	-	-	-	-	-	-	-	*Ahrensia kielensis*
	L-1-12	-	rod	-	-	O	M	+	+	+	+	-	-	-	+	+	-	+	+	+	-	+	+	-	-	-	-	-	orange	*Pseudoalteromonas marina*
	L-1-13	-	rod	-	-	O	M	+	+	+	+	-	-	-	+	-	-	+	+	+	-	+	+	-	-	-	-	-	-	*Pseudoalteromonas elyakovii*
	L-1-14	-	rod	+	polar	O	M	+	+	+	+	-	-	-	+	+	-	+	+	+	-	+	+	+	-	-	-	-	-	*Pseudoalteromonas marina*
	L-1-15	-	rod	-	-	O	M	+	+	+	+	-	-	-	+	+	-	+	+	+	-	+	+	+	-	-	-	-	-	*Pseudoalteromonas marina*
	L-1-16	-	rod	-	-	O	M	+	+	-	-	-	-	-	-	-	-	+	+	+	-	+	+	-	-	-	-	-	-	*Psychrobacter* sp.
	L-1-17	-	rod	+	polar	F	M	+	+	+	-	-	-	-	+	+	-	+	+	+	-	+	+	-	-	-	-	-	-	*Vibrio cyclitrophicus*
	L-1-18	-	rod	-	-	O	M	+	+	+	+	-	-	-	-	-	-	+	+	-	-	+	+	+	-	-	-	-	-	*Shewanella frigidimarina*
	L-1-19	-	rod	-	-	O	M	+	+	+	-	-	-	-	+	+	-	+	+	+	-	+	+	+	-	-	-	-	-	*Pseudoalteromonas marina*
	L-1-20	-	rod	-	-	O	M	+	+	+	+	-	-	-	+	+	-	+	+	+	-	+	+	-	-	-	-	-	-	*Pseudoalteromonas marina*
	L-1-21	-	rod	-	-	O	M	+	+	+	-	-	-	-	-	-	-	+	+	+	-	+	+	-	-	-	-	-	-	*Pseudoalteromonas* sp.
	L-1-22	-	rod	-	-	O	M	+	+	+	-	-	-	-	+	+	-	+	+	+	-	+	+	+	-	-	-	-	-	*Pseudoalteromonas marina*
	L-1-23	-	rod	+	polar	O	M	+	+	+	-	-	-	-	-	-	-	+	+	-	-	+	+	-	-	-	-	-	-	*Shewanella frigidimarina*
	L-1-24	-	rod	-	-	O	M	+	+	+	+	-	-	-	+	+	-	+	+	+	-	+	+	-	-	-	-	-	-	*Pseudoalteromonas marina*
	L-1-25	-	rod	-	-	O	M	+	+	+	-	-	-	-	+	+	-	+	+	+	-	+	+	-	-	-	-	-	-	*Pseudoalteromonas marina*
	L-1-26	-	rod	-	-	O	M	+	+	-	+	-	-	-	-	+	-	+	+	-	-	+	+	-	-	-	-	-	-	*Winogradskyella thalassocola*
	L-1-27	-	rod	+	polar	O	M	+	+	+	-	-	-	-	-	-	-	+	+	-	-	+	+	+	-	-	-	+	-	*Pseudoalteromonas marina*
	L-1-28	-	rod	-	-	O	M	+	+	+	+	-	-	-	+	+	-	+	+	+	-	+	+	+	-	-	-	-	-	*Lacinutrix* sp.
	L-1-29	-	rod	+	polar	F	M	+	+	+	+	-	-	-	+	-	-	+	+	-	-	+	+	+	-	-	-	-	-	*Vibrio splendidus*

1 Table S1. Continued.

Origin	Strain	Gram stain	Morphology	Motility	Flagellum	OF test	Na^+ requirement	Oxidase	Catalase	DNase	Gelatinase	Agarase	Luminescence	Spore formation	Caseinase	Amylase	Growth at 4 degC	Growth at 15 degC	Growth at 25 degC	Growth at 30 degC	Growth at 37 degC	Lipase (Tween20)	Lipase (Tween80)	Alginase	Fucoidanase	Chitinase	Elastinase	Growth on TCBS	Pigmentation	Taxa
Li-2	L-2-1	-	rod	-	-	F	T	+	+	-	-	-	-	-	+	-	-	+	+	+	-	+	+	-	-	-	-	+	-	*Pseudomonas* sp.
	L-2-2	-	rod	-	-	F	T	+	+	-	-	-	-	-	+	-	-	+	+	+	-	+	+	-	-	-	-	+	-	*Pseudomonas* sp.
	L-2-3	-	rod	-	-	O	M	+	+	-	-	-	-	-	-	-	-	+	+	-	-	+	+	-	-	-	-	-	-	*Roseobacter* sp.
	L-2-4	-	rod	-	-	O	M	+	+	-	-	-	-	-	-	-	-	+	+	-	-	+	+	-	-	-	-	-	-	*Roseobacter* sp.
	L-2-5	-	coccus	-	-	O	M	+	+	-	-	-	-	-	-	-	-	+	+	-	-	+	+	-	-	-	-	-	-	*Roseobacter* sp.
	L-2-6	-	rod	-	-	F	M	+	+	+	-	-	-	-	+	-	-	+	+	+	-	+	+	-	-	-	-	+	-	*Vibrio halioticoli*
	L-2-7	-	rod	-	-	O	M	+	+	-	-	-	-	-	-	-	-	+	+	-	-	+	+	-	-	-	-	-	-	*Roseobacter* sp.
	L-2-8	-	rod	-	-	F	M	+	+	+	-	+	-	-	+	-	-	+	+	-	-	+	+	-	-	-	-	+	brown	*Shewanella baltica*
	L-2-9	-	rod	-	-	O	T	+	+	-	-	-	-	-	+	-	-	+	+	+	-	+	+	-	-	-	-	+	-	*Pseudomonas* sp.
	L-2-10	-	rod	-	-	O	M	+	+	-	-	-	-	-	-	-	-	+	+	-	-	+	+	-	-	-	-	-	-	*Roseobacter* sp.
	L-2-11	-	rod	-	-	O	T	+	+	-	-	-	-	-	+	-	-	+	+	+	-	+	+	-	-	-	-	-	-	*Roseobacter* sp.
	L-2-12	-	rod	-	-	O	M	+	+	-	-	-	-	-	-	-	-	+	+	-	-	+	+	-	-	-	-	-	-	*Roseobacter* sp.
	L-2-13	-	rod	-	-	O	M	+	+	-	-	-	-	-	-	-	-	+	+	-	-	+	+	-	-	-	-	-	-	*Roseobacter* sp.
	L-2-14	-	rod	-	-	O	M	+	+	+	+	-	-	-	+	-	-	+	+	+	-	+	+	-	-	-	-	-	-	*Agrobacterium* sp.
	L-2-15	-	rod	-	-	O	M	+	+	-	-	-	-	-	-	-	-	+	+	-	-	+	+	-	-	-	-	-	-	*Roseobacter* sp.
	L-2-16	-	rod	-	-	O	M	+	+	-	+	-	-	-	-	-	-	+	+	+	-	+	+	-	-	-	-	-	-	*Roseobacter* sp.
	L-2-17	-	rod	-	-	O	M	+	+	-	-	-	-	-	-	-	-	+	+	-	-	+	+	-	-	-	-	-	-	*Roseobacter* sp.
	L-2-18	-	rod	-	-	O	M	+	+	-	-	-	-	-	-	-	-	+	+	-	-	+	+	-	-	-	-	-	-	*Roseobacter* sp.
	L-2-19	-	rod	+	polar	F	M	+	+	+	-	-	-	-	-	+	-	+	+	+	-	+	+	-	-	-	-	+	-	*Vibrio halioticoli*
	L-2-20	-	rod	-	-	O	M	+	+	-	-	-	-	-	-	-	-	+	+	-	-	+	+	-	-	-	-	-	-	*Roseobacter sp.*
	L-2-21	-	rod	-	-	F	M	+	+	+	+	+	-	-	+	-	-	+	+	+	-	+	+	-	-	-	-	+	-	*Vibrio galicus*
	L-2-22	-	rod	-	-	F	M	+	+	+	+	+	-	-	+	-	-	+	+	+	-	+	+	-	-	-	-	+	-	*Vibrio galicus*
	L-2-23	-	rod	-	-	O	M	+	+	-	+	-	-	-	-	-	-	+	+	+	-	+	+	-	-	-	-	-	-	*Roseobacter* sp.
	L-2-24	-	rod	-	-	O	M	+	+	-	-	-	-	-	-	-	-	+	+	-	-	+	+	-	-	-	-	-	-	*Roseobacter* sp.
	L-2-25	-	rod	-	-	O	M	+	+	-	-	-	-	-	-	-	-	+	+	-	-	+	+	-	-	-	-	-	-	*Roseobacter* sp.
	L-2-26	-	rod	-	-	O	M	+	+	-	-	-	-	-	-	-	-	+	+	-	-	+	+	-	-	-	-	-	-	*Roseobacter* sp.
	L-2-27	-	rod	-	-	O	M	+	+	-	-	-	-	-	-	-	-	+	+	-	-	+	+	-	-	-	-	-	-	*Roseobacter* sp.
	L-2-28	-	rod	-	-	O	M	+	+	+	+	-	-	-	-	-	-	+	+	-	-	+	+	-	-	-	-	-	brown	*Roseobacter* sp.
	L-2-29	-	rod	-	-	O	M	+	+	-	-	-	-	-	-	-	-	+	+	-	-	+	+	-	-	-	-	-	-	*Roseobacter* sp.
	L-2-30	-	rod	-	-	F	M	+	+	+	-	-	-	-	-	+	-	+	+	+	-	+	+	-	-	-	-	+	-	*Vibrio halioticoli*

2

Table S1. Continued.

Origin	Strain	Gram stain	Morphology	Motility	Flagellum	OF test	Na^+ requirement	Oxidase	Catalase	DNase	Gelatinase	Agarase	Luminescence	Spore formation	Caseinase	Amylase	Growth at 4 degC	Growth at 15 degC	Growth at 25 degC	Growth at 30 degC	Growth at 37 degC	Lipase (Tween20)	Lipase (Tween80)	Alginase	Fucoidanase	Chitinase	Elastinase	Growth on TCBS	Pigmentation	Taxa
C-1	B-1-1	-	rod	-	-	O	M	+	+	-	-	-	-	-	-	-	-	+	+	-	-	+	+	-	-	-	-	-	orange	*Psychroserpens* sp.
	B-1-2	-	rod	-	-	O	M	+	+	-	+	+	-	-	-	-	-	+	+	+	-	+	+	-	-	-	-	-	orange	*Cytophaga* sp.
	B-1-3	-	coccus	-	-	F	M	+	+	-	+	-	-	-	-	+	-	+	+	-	-	+	+	-	-	-	-	-	orange	*Winogradskyella thalassocola*
	B-1-4	-	rod	-	-	O	M	+	+	-	+	-	-	-	+	-	-	+	+	-	-	+	+	-	-	-	-	-	orange	*Bacteroidetes* bacterium
	B-1-5	-	coccus	-	-	O	M	+	+	+	+	-	-	-	+	-	-	+	+	-	-	+	+	-	-	-	-	-	orange	*Bacteroidetes* bacterium
	B-1-6	-	rod	-	-	O	M	+	+	+	+	-	-	-	+	-	-	+	+	+	-	+	+	-	-	-	-	-	orange	*Cytophaga* sp.
	B-1-7	-	rod	-	-	F	M	+	+	+	+	-	-	-	+	+	-	+	+	+	-	+	+	-	-	-	-	-	orange	*Zobellia amurskyensis*
	B-1-8	-	rod	-	-	O	M	+	+	+	-	-	-	-	-	-	-	+	+	+	-	+	+	-	-	-	-	-	orange	*Verrucomicrobia* bacterium
	B-1-9	-	coccus	-	-	O	M	+	+	-	+	-	-	-	-	-	-	+	+	+	-	+	+	-	-	-	-	-	orange	*Maribacter aquivivus*.
	B-1-10	-	rod	-	-	O	M	+	+	+	+	-	-	-	-	-	-	+	+	+	-	+	+	-	-	-	-	-	-	*Octadecabacter* sp.
	B-1-11	-	rod	-	-	O	M	+	+	+	+	-	-	-	+	-	-	+	+	+	-	+	+	-	-	-	-	-	-	*Pseudoalteromonas* sp.
	B-1-12	-	rod	-	-	F	M	+	+	+	+	-	-	-	-	-	-	+	+	+	-	+	+	-	-	-	-	-	orange	*Lacinutrix* sp.
	B-1-13	-	rod	-	-	O	M	+	+	+	+	-	-	-	+	+	-	+	+	+	-	+	+	-	-	-	-	-	-	*Pseudoalteromonas marina*
	B-1-14	-	rod	-	-	O	M	+	+	+	-	-	-	-	+	-	-	+	+	+	-	+	+	-	-	-	-	-	-	*Pseudoalteromonas* sp.
	B-1-15	-	rod	-	-	O	M	+	+	-	+	-	-	-	-	-	-	+	+	+	-	+	+	-	-	-	-	-	-	*Acidovorax* sp.
	B-1-16	-	rod	-	-	O	M	+	+	+	+	-	-	-	+	+	-	+	+	+	-	+	+	-	-	-	-	-	-	*Pseudoalteromonas marina*
	B-1-17	-	rod	-	-	O	M	+	+	+	-	-	-	-	+	-	-	+	+	+	-	+	+	-	-	-	-	-	orange	*Lacinutrix* sp.
	B-1-18	-	rod	-	-	O	M	+	+	-	-	-	-	-	-	-	-	+	+	+	-	+	+	-	-	-	-	-	orange	*Sphingomonas* sp.
	B-1-19	-	coccus	-	-	F	M	+	+	-	-	-	-	-	-	-	-	+	+	+	-	-	-	-	-	-	-	-	-	*Rothia amarae*
	B-1-20	-	rod	-	-	O	M	+	+	-	+	-	-	-	-	+	-	+	+	-	-	+	+	-	-	-	-	-	orange	*Lacinutrix* sp.
	B-1-21	-	rod	-	-	O	M	+	+	+	+	-	-	-	+	-	-	+	+	+	-	+	+	-	-	-	-	-	-	*Pseudoalteromonas elyakovii*
	B-1-22	-	rod	-	-	O	M	+	+	+	+	-	-	-	+	+	-	+	+	+	-	+	+	-	-	-	-	-	-	*Pseudoalteromonas marina*
	B-1-23	-	rod	-	-	O	M	+	+	+	+	-	-	-	+	-	-	+	+	-	-	+	+	-	-	-	-	-	orange	*Lacinutrix* sp.
	B-1-24	-	rod	-	-	O	M	+	+	+	+	-	-	-	-	-	-	+	+	+	-	+	+	-	-	-	-	-	-	*Williamsia muralis*
	B-1-25	-	rod	+	polar	O	M	+	+	+	+	-	-	-	+	+	-	+	+	+	-	+	+	-	-	-	-	-	-	*Pseudoalteromonas elyakovii*
	B-1-26	-	rod	-	-	O	M	+	+	+	+	-	-	-	+	-	-	+	+	+	-	+	+	-	-	-	-	-	-	*Pseudoalteromonas* sp.
	B-1-27	-	rod	+	polar	O	M	+	+	+	+	-	-	-	+	-	-	+	+	+	-	+	+	-	-	-	-	-	-	*Pseudoalteromonas elyakovii*
	B-1-28	-	rod	-	-	O	M	+	+	+	+	-	-	-	+	+	-	+	+	+	-	+	+	+	-	-	-	-	-	*Pseudoalteromonas marina*
	B-1-30	-	rod	+	polar	O	M	+	+	+	+	-	-	-	+	-	-	+	+	+	-	+	+	-	-	+	-	-	-	*Pseudoalteromonas elyakovii*

Table S1. Continued.

Origin	Strain	Gram stain	Morphology	Motility	Flagellum	OF test	Na^+ requirement	Oxidase	Catalase	DNase	Gelatinase	Agarase	Luminescence	Spore formation	Caseinase	Amylase	Growth at 4 degC	Growth at 15 degC	Growth at 25 degC	Growth at 30 degC	Growth at 37 degC	Lipase (Tween20)	Lipase (Tween80)	Alginase	Fucoidanase	Chitinase	Elastinase	Growth on TCBS	Pigmentation	Taxa
C-2	B-2-1	-	rod	-	-	N	M	+	-	-	+	-	-	-	-	-	-	+	+	-	-	+	+	-	-	-	-	-	yellow	*Psychroserpens burtonensis*
	B-2-2	-	coccus	-	-	O	M	+	+	+	+	+	-	-	+	-	-	+	+	+	-	+	+	-	-	-	-	-	-	*Pseudoalteromonas elyakovii*
	B-2-3	-	coccus	-	-	F	M	+	-	+	+	+	-	-	+	+	-	+	+	-	-	+	+	-	-	-	-	+	brown	*Shewanella baltica*
	B-2-4	-	rod	-	-	O	M	+	+	+	+	-	-	-	+	+	-	+	+	+	-	+	+	-	-	-	-	-	-	*Pseudoalteromonas elyakovii*
	B-2-5	-	rod	-	-	N	M	+	-	-	-	-	-	-	-	-	-	+	+	-	-	+	+	-	-	-	-	-	-	*Roseobacter* sp.
	B-2-6	-	rod	+	polar	O	M	+	+	+	+	-	-	-	+	-	-	+	+	+	-	+	+	-	-	-	-	-	-	*Pseudoalteromonas elyakovii*
	B-2-7	-	rod	-	-	O	T	+	+	-	-	-	-	-	-	-	-	+	+	+	-	+	+	-	-	-	-	-	-	*Psychrobacter okhotskensis*
	B-2-8	+	rod	+	polar	F	H	+	+	+	+	-	-	-	+	+	-	+	+	-	-	+	+	-	-	+	-	-	-	*Vibrio tasmaniensis*
	B-2-9	-	rod	-	-	F	H	+	+	+	+	-	-	-	+	+	-	+	+	-	-	+	+	-	-	+	-	-	-	*Vibrio splendidus*
	B-2-11	-	rod	+	polar	F	M	+	+	+	+	-	-	-	+	+	-	+	+	-	-	+	+	-	-	+	-	-	-	*Vibrio splendidus*
	B-2-12	-	rod	-	-	O	M	+	+	+	+	+	-	-	+	-	-	+	+	+	-	+	+	-	-	-	-	-	-	*Pseudoalteromonas elyakovii*
	B-2-13	-	rod	-	-	F	H	+	+	+	+	-	-	-	+	+	-	+	+	-	-	+	+	-	-	+	-	+	-	*Vibrio splendidus*
	B-2-14	-	rod	-	-	O	M	+	+	+	+	-	-	-	+	-	-	+	+	+	-	+	+	-	-	-	-	-	-	*Pseudoalteromonas elyakovii*
	B-2-15	-	rod	-	-	O	M	+	-	+	+	+	-	-	+	+	-	+	+	-	-	+	+	-	-	-	-	+	brown	*Shewanella baltica*
	B-2-16	-	coccus	-	-	O	M	+	+	+	+	-	-	-	+	+	-	+	+	+	-	+	+	-	-	-	-	-	-	*Pseudoalteromonas elyakovii*
	B-2-17	-	rod	-	-	O	M	+	-	+	+	+	-	-	+	+	-	+	+	-	-	+	+	+	-	-	-	+	brown	*Shewanella baltica*
	B-2-18	-	rod	-	-	F	H	+	+	+	+	-	-	-	+	+	-	+	+	-	-	+	+	-	-	+	-	+	-	*Vibrio splendidus*
	B-2-19	-	rod	-	-	F	M	+	+	+	+	+	-	-	+	-	-	+	+	+	-	+	+	+	-	-	-	-	-	*Vibrio splendidus*
	B-2-20	-	rod	-	-	O	M	+	-	-	+	-	-	-	-	-	-	+	+	-	-	+	+	-	-	-	-	-	yellow	*Pseudoalteromonas burtonensis*
	B-2-21	-	rod	-	-	O	M	+	+	+	+	-	-	-	+	+	-	+	+	+	-	+	+	-	-	-	-	-	-	*Pseudoalteromonas elyakovii*
	B-2-22	-	rod	-	-	O	M	+	+	+	+	-	-	-	+	+	-	+	+	+	-	+	+	-	-	-	-	-	-	*Pseudoalteromonas arctica*
	B-2-23	-	rod	-	-	O	M	+	+	+	+	+	-	-	+	-	-	+	+	+	-	+	+	-	-	-	-	-	-	*Pseudoalteromonas elyakovii*
	B-2-24	-	rod	-	-	O	M	+	+	+	+	+	-	-	+	-	-	+	+	+	-	+	+	-	-	-	-	-	-	*Pseudoalteromonas elyakovii*
	B-2-25	-	coccus	+	polar	F	H	+	+	+	+	-	-	-	+	+	-	+	+	-	-	+	+	-	-	+	-	+	-	*Vibrio tasmaniensis*
	B-2-26	-	rod	-	-	O	M	+	+	+	+	+	-	-	+	-	-	+	+	+	-	+	+	-	-	-	-	-	-	*Pseudoalteromonas elyakovii*
	B-2-27	-	rod	-	-	F	M	+	-	+	+	+	-	-	+	+	-	+	+	-	-	+	+	-	-	-	-	+	brown	*Shewanella baltica*
	B-2-28	-	rod	-	-	O	H	+	+	+	+	-	-	-	+	+	-	+	+	-	-	+	+	-	-	+	-	+	-	*Vibrio splendidus*
	B-2-29	-	rod	-	-	O	M	+	+	+	+	-	-	-	+	+	-	+	+	+	-	+	+	-	-	-	-	-	-	*Pseudoalteromonas elyakovii*
	B-2-30	-	rod	-	-	O	M	+	-	+	+	-	-	-	+	-	-	+	+	+	-	+	+	-	-	-	-	-	-	*Pseudoalteromonas elyakovii*

Table S1. Continued.

Origin	Strain	Gram stain	Morphology	Motility	Flagellum	OF test	Na^+ requirement	Oxidase	Catalase	DNase	Gelatinase	Agarase	Luminescence	Spore formation	Caseinase	Amylase	Growth at 4 degC	Growth at 15 degC	Growth at 25 degC	Growth at 30 degC	Growth at 37 degC	Lipase (Tween20)	Lipase (Tween80)	Alginase	Fucoidanase	Chitinase	Elastinase	Growth on TCBS	Pigmentation	Taxa
U-1	U-1-1	-	rod	+	polar	O	M	+	+	+	+	-	-	-	+	-	-	+	+	+	-	+	+	-	-	-	-	-	-	*Pseudoalteromonas arctica*
	U-1-2	-	rod	+	polar	O	M	+	+	+	-	-	-	-	+	-	-	+	+	+	-	+	+	-	-	-	-	-	-	*Pseudoalteromonas arctica*
	U-1-3	-	rod	-	-	O	H	+	+	+	-	-	-	-	+	-	-	+	+	+	-	+	+	-	-	-	-	-	-	*Pseudoalteromonas elyakovii*
	U-1-4	-	rod	+	polar	O	M	+	+	+	+	-	-	-	+	-	-	+	+	+	-	+	+	-	-	-	-	-	-	*Pseudoalteromonas elyakovii*
	U-1-5	-	rod	-	-	O	M	+	+	-	-	-	-	-	-	-	-	+	+	-	-	+	+	-	-	-	-	-	orange	*Bacteroidetes* bacterium
	U-1-6	-	rod	-	-	O	M	+	+	+	+	-	-	-	+	-	-	+	+	+	-	+	+	-	-	-	-	-	-	*Pseudoalteromonas elyakovii*
	U-1-7	-	rod	-	-	O	H	+	+	+	+	-	-	-	+	-	-	+	+	+	-	+	+	-	-	-	-	-	-	*Pseudoalteromonas elyakovii*
	U-1-9	-	rod	-	-	O	M	+	+	+	+	-	-	-	+	-	-	+	+	+	-	+	+	-	-	-	-	-	-	*Pseudoalteromonas elyakovii*
	U-1-10	-	rod	-	-	O	M	+	+	+	+	+	-	-	+	-	-	+	+	+	-	+	+	-	-	-	-	-	-	*Psychroserpens burtonensis*
	U-1-11	-	rod	-	-	O	M	+	+	+	-	-	-	-	-	+	-	+	+	-	-	+	+	-	-	-	-	-	cream	*Psychroserpens burtonensis*
	U-1-12	-	rod	-	-	F	M	+	+	+	+	-	-	-	-	+	-	+	+	-	-	+	+	-	-	-	-	-	orange	*Winogradskyella thalassocola*
	U-1-13	-	rod	-	-	F	H	+	+	+	+	-	-	-	-	-	-	+	+	+	-	+	+	-	-	-	-	-	orange	*Alteromonas* sp.
	U-1-14	-	rod	-	-	O	H	+	+	-	-	-	-	-	-	-	-	+	+	-	-	+	-	-	-	-	-	-	-	*Marinosulfonomonas methylotropha*
	U-1-15	-	rod	+	polar	O	M	+	+	+	+	-	-	-	+	-	-	+	+	+	-	+	+	-	-	-	-	-	-	*Pseudoalteromonas elyakovii*
	U-1-16	-	rod	+	polar	O	M	+	+	+	+	-	-	-	+	-	-	+	+	+	-	+	+	-	-	-	-	-	-	*Pseudoalteromonas arctica*
	U-1-18	-	rod	-	-	O	M	+	+	-	-	-	-	-	-	-	-	+	+	+	-	-	-	-	-	-	-	-	-	*Psychrobacter* sp.
	U-1-19	-	rod	-	-	O	M	+	+	-	-	-	-	+	-	-	-	+	+	-	-	+	+	-	-	-	-	-	-	*Psychrobacter* sp.
	U-1-20	-	rod	-	-	O	M	+	+	+	+	-	-	-	+	+	-	+	+	+	-	+	+	-	-	-	-	-	-	*Pseudoalteromonas elyakovii*
	U-1-21	-	rod	-	-	F	M	+	-	+	+	-	-	-	-	+	-	+	+	+	-	+	+	-	-	-	-	-	orange	*Winogradskyella eximia*
	U-1-22	-	rod	+	polar	O	M	+	+	+	+	-	-	-	+	-	-	+	+	+	-	+	+	-	-	-	-	-	-	*Pseudoalteromonas elyakovii*
	U-1-23	-	rod	+	polar	O	M	+	+	+	+	-	-	-	+	-	-	+	+	+	-	+	+	-	-	-	-	-	-	*Pseudoalteromonas elyakovii*
	U-1-24	-	rod	-	-	O	M	+	+	+	+	-	-	-	+	+	-	+	+	+	-	+	+	-	-	-	-	-	-	*Pseudoalteromonas marina*
	U-1-25	-	rod	+	polar	O	M	+	+	+	+	-	-	+	+	-	-	+	+	+	-	+	+	-	-	-	-	-	-	*Pseudoalteromonas elyakovii*
	U-1-26	-	rod	-	-	O	H	+	+	+	+	-	-	-	+	-	-	+	+	+	-	+	+	-	-	-	-	-	-	*Pseudoalteromonas elyakovii*
	U-1-27	-	rod	-	-	O	M	+	+	+	-	-	-	-	-	-	-	+	+	-	-	+	+	-	-	-	-	-	yellow	*Lacinutrix* sp.
	U-1-28	-	rod	-	-	O	M	+	+	+	+	-	-	-	-	+	-	+	+	-	-	+	+	-	-	-	-	-	orange	*Psychroserpens* sp.
	U-1-29	-	rod	+	polar	O	H	+	+	+	+	-	-	-	+	+	-	+	+	-	-	+	+	-	-	+	+	-	-	*Pseudoalteromonas elyakovii*
	U-1-30	-	rod	+	polar	O	M	+	+	+	+	-	-	-	+	-	-	+	+	+	-	+	+	-	-	-	-	-	-	*Pseudoalteromonas arctica*

1 Table S1. Continued.

Origin	Strain	Gram stain	Morphology	Motility	Flagellum	OF test	Na^+ requirement	Oxidase	Catalase	DNase	Gelatinase	Agarase	Luminescence	Spore formation	Caseinase	Amylase	Growth at 4 degC	Growth at 15 degC	Growth at 25 degC	Growth at 30 degC	Growth at 37 degC	Lipase (Tween20)	Lipase (Tween80)	Alginase	Fucoidanase	Chitinase	Elastinase	Growth on TCBS	Pigmentation	Taxa
U-2	U-2-1	-	rod	-	-	O	M	+	+	+	-	+	-	-	+	+	-	+	+	+	-	+	+	-	-	-	+	-	-	*Pseudoalteromonas elyakovii*
	U-2-2	-	rod	-	-	O	M	+	+	+	-	-	-	-	-	+	-	+	+	-	-	+	+	-	-	-	-	-	yellow	*Lacinutrix* sp.
	U-2-3	-	rod	-	-	O	M	+	+	+	-	-	-	-	+	+	-	+	+	-	-	+	+	-	-	-	-	-	yellow	*Ulvibacter* sp.
	U-2-4	-	rod	-	-	O	M	+	+	+	-	-	-	-	-	+	-	+	+	-	-	+	+	-	-	-	-	-	-	*Roseobacter* sp.
	U-2-5	-	rod	-	-	O	M	+	+	+	-	+	-	-	+	+	-	+	+	+	-	+	+	-	-	-	+	-	-	*Pseudoalteromonas elyakovii*
	U-2-6	-	rod	+	polar	O	M	+	+	+	-	+	-	-	+	-	-	+	+	+	-	+	+	-	-	-	+	-	-	*Pseudoalteromonas elyakovii*
	U-2-7	-	rod	-	-	O	M	+	+	-	+	-	-	-	-	+	-	+	+	-	-	+	+	-	-	-	-	-	yellow	*Psychroserpens burtonensis*
	U-2-8	-	rod	-	-	O	M	+	+	-	+	+	-	-	+	+	-	+	+	-	-	+	+	-	-	-	-	-	yellow	*Pseudoalteromonas prydzensis*
	U-2-9	-	rod	-	-	O	M	+	+	+	-	-	-	-	-	+	-	+	+	-	-	+	+	-	-	-	-	-	cream	*Roseobacter* sp.
	U-2-10	-	rod	-	-	F	M	+	+	+	-	+	-	-	+	-	-	+	+	+	-	+	+	-	-	-	+	-	-	*Pseudoalteromonas elyakovii*
	U-2-11	-	rod	+	polar	F	M	+	+	+	-	-	-	-	-	+	-	+	-	-	-	+	+	-	-	+	-	+	-	*Aliivibrio logei*
	U-2-13	-	rod	-	-	O	M	+	+	+	-	-	-	-	-	+	-	+	+	+	-	+	+	-	-	-	+	-	cream	*Colwellia* sp.
	U-2-14	-	rod	-	-	O	M	+	+	-	-	+	-	-	+	-	-	+	+	+	-	+	+	-	-	-	+	-	-	*Pseudoalteromonas elyakovii*
	U-2-15	-	rod	-	-	O	M	+	+	+	-	-	-	-	-	+	-	+	+	-	-	+	+	-	-	-	-	-	-	*Roseobacter* sp.
	U-2-16	-	rod	-	-	O	M	+	+	+	-	-	-	-	-	+	-	+	+	+	-	+	+	-	-	-	+	-	-	*Pseudoalteromonas elyakovii*
	U-2-17	-	rod	+	polar	O	M	+	+	+	-	-	-	-	+	-	-	+	+	+	-	+	+	-	-	-	+	-	-	*Pseudoalteromonas elyakovii*
	U-2-19	-	rod	+	polar	O	M	+	+	+	-	+	-	-	+	-	-	+	+	+	-	+	+	-	-	-	+	-	-	*Pseudoalteromonas elyakovii*
	U-2-20	-	rod	+	polar	F	M	+	+	+	-	-	-	-	-	-	-	+	+	-	-	+	+	-	-	-	-	+	-	*Vibrio splendidus*
	U-2-21	-	rod	+	polar	O	M	+	+	+	+	+	-	-	+	-	-	+	+	+	-	+	+	-	-	-	+	-	-	*Pseudoalteromonas elyakovii*
	U-2-22	-	rod	-	-	O	M	+	+	+	+	-	-	-	-	-	-	+	+	+	-	+	+	-	-	-	+	-	-	*Pseudoalteromonas elyakovii*
	U-2-23	-	rod	-	-	F	M	+	+	+	-	-	-	-	-	+	-	+	+	-	-	+	+	-	-	-	-	-	-	*Psychromonas arctica*
	U-2-24	-	rod	-	-	O	M	+	+	-	-	-	-	-	-	+	-	+	+	-	-	+	+	-	-	-	-	-	-	*Verrucomicrobia* bacterium
	U-2-26	-	rod	-	-	F	M	+	+	+	-	-	-	-	-	+	-	+	+	-	-	+	+	-	-	-	-	-	-	*Psychromonas arctica*
	U-2-27	-	rod	-	-	O	M	+	+	+	-	+	-	-	+	-	-	+	+	+	-	+	+	-	-	-	+	-	-	*Pseudoalteromonas elyakovii*
	U-2-28	-	rod	-	-	O	M	+	+	+	-	+	-	-	-	-	-	+	+	+	-	+	+	-	-	-	+	-	-	*Pseudoalteromonas elyakovii*
	U-2-29	-	rod	+	-	O	M	+	+	+	-	-	-	-	+	-	-	+	+	+	-	+	+	-	-	-	-	-	-	*Pseudoalteromonas elyakovii*
2	U-2-30	-	rod	-	-	O	M	+	+	+	-	+	-	-	+	+	-	+	+	+	-	+	+	-	-	-	+	-	-	*Pseudoalteromonas elyakovii*

Table S2. Phylotype analysis of bacterial 16S rRNA gene clone sequences recovered from holothurian samples. See Fig. 2 for PCR primers.

Represent-ative clones	No. of clones: Small intestine		No. of clones: Large intestine		No. of clones: Body surface		No. of clones: Coelomic fluid		Taxa	Closest relative (accession no.)	%
	Si-09	Si-40	Li-09	Li-40	U-09	U-40	C-09	C-40			
S-09-4	7	10	2	5	0	0	0	0	*Proteobacteria; Alphaproteobacteria; Rhodobacterales; Rhodobacteraceae; Roseobacter* clade	Uncultured alpha-proteobacterium clone G3-55 (EU005317)	99
L-40-35	0	0	0	1	0	0	0	0	*Proteobacteria; Alphaproteobacteria; Rhodobacterales; Rhodobacteraceae; Roseobacter* clade	Uncultured bacterium clone aquased47 (DQ028264)	97
L-40-8	0	0	0	1	0	0	0	0	*Proteobacteria; Alphaproteobacteria; Rhodobacterales; Rhodobacteraceae;*	Uncultured bacterium clone EPR4059-B2Bc5 (EU491552)	96
L-09-38	0	0	1	0	0	0	1	1	*Proteobacteria; Alphaproteobacteria;*	Uncultured alpha-proteobacterium clone UA02 (DQ269055)	96
U-09-2	0	0	0	0	12	12	5	1	*Proteobacteria; Alphaproteobacteria; Rhodospirillales; Rhodospirillaceae; Defluvicoccus*	Uncultured alpha proteobacterium clone SHBC 1149 (GQ350217)	98
B-09-325	0	0	0	0	0	0	1	0	*Proteobacteria; Alphaproteobacteria; Rhodospirillales; Rhodospirillaceae; Defluvicoccus*	Uncultured bacterium clone NED5E9 (EF445270)	84
L-40-5	0	0	0	1	0	0	0	0	*Proteobacteria; Alphaproteobacteria; Rhodospirillales; Rhodospirillaceae*	*Rhodospirillaceae* bacterium CL-UU02 (DQ401091)	99
S-40-29	0	1	0	0	0	0	0	0	*Proteobacteria; Alphaproteobacteria; Rhizobiales; Hyphomicrobiaceae*	Uncultured bacterium clone SHFH697 (FJ203608)	98
B-09-288	0	0	0	0	0	0	6	7	*Proteobacteria; Alphaproteobacteria; Rickettsiales*	Uncultured bacterium clone ELB16-042 (DQ015803)	82
B-09-280	0	0	0	0	0	0	1	0	*Proteobacteria; Alphaproteobacteria; Rickettsiales*	Uncultured bacterium clone RB90b-112 (AM159487)	96
B-09-239	0	0	0	0	0	0	1	0	*Proteobacteria; Alphaproteobacteria; Rickettsiales*	Uncultured alpha-proteobacterium clone Z-1-211 (FJ666148)	92
B-09-343	0	0	0	0	0	0	1	0	*Proteobacteria; Alphaproteobacteria; Rickettsiales*	Uncultured bacterium clone S25_780 (EF574436)	85
S-09-17	2	2	1	0	0	4	2	0	*Proteobacteria; Betaproteobacteria; Burkholderiales; Comamonadaceae; Delftia*	Uncultured bacterium clone nbt84f11 (EU538302)	99
U-09-128	0	0	0	0	1	0	0	0	*Proteobacteria; Gammaproteobacteria; Vibrionales; Vibrionaceae; Vibrio*	Uncultured bacterium clone A017_NCI (FJ456765)	99
L-40-7	0	0	0	1	0	0	0	0	*Proteobacteria; Gammaproteobacteria; Thiotrichales; Piscirickettsiaceae*	Uncultured bacterium clone TfC20L41 (EU362305)	99
B-09-301	0	0	0	0	0	0	1	0	*Proteobacteria; Gammaproteobacteria; Thiotrichales*	Uncultured gamma proteobacterium clone DS095 (DQ234178)	95
S-40-4	0	2	3	0	0	0	0	0	*Proteobacteria; Deltaproteobacteria; Desulfobacterales; Desulfobulbaceae*	Uncultured delta proteobacterium clone KorMud-V8C76	97
B-40-44	0	0	0	0	0	0	0	1	*Proteobacteira; Epsilonproteobacteria*	Uncultured *Bathymodiolus platifrons* gill symbiont (AB250697)	92
B-40-18	0	0	0	0	0	0	2	12	*Proteobacteira; Epsilonproteobacteria; Campylobacterales; Campylobacteraceae; Sulfurospirillum*	Uncultured bacterium clone FS140-2B-02 (AY704396)	92

Table S2. continued

Represent-ative clones	No. of clones								Taxa	Closest sequence (accession no.)	%
	Small intestine		Large intestine		Body surface		Coelomic fluid				
	Si-09	Si-40	Li-09	Li-40	U-09	U-40	C-09	C-40			
S-09-11	3	1	2	1	0	0	0	0	*Planctomycetes; Planctomycetacia; Planctomycetales; Planctomycetaceae; Pirellula*	Uncultured *Planctomycete* clone Belgica2005/10-ZG-18 (DQ351810)	96
L-09-6	0	0	1	0	0	0	0	0	*Planctomycetes; Planctomycetacia; Planctomycetales; Planctomycetaceae; Pirellula*	Uncultured *planctomycete* clone 3m04AISD8R	93
L-09-25	0	0	1	0	0	0	0	0	*Planctomycetes; Planctomycetacia; Planctomycetales; Planctomycetaceae; Pirellula*	Uncultured bacterium clone CBM01C11 (EF395637)	95
S-09-29	1	0	0	0	0	0	0	0	*Planctomycetes; Planctomycetacia; Planctomycetales; Planctomycetaceae; Pirellula*	Uncultured bacterium clone SHFH596 (FJ203525)	97
L-09-12	0	0	1	0	0	0	0	0	*Planctomycetes; Planctomycetacia; Planctomycetales; Planctomycetaceae; Pirellula*	Uncultured bacterium clone SHFH395 (FJ203368)	94
S-09-8	1	0	0	1	0	0	0	0	*Planctomycetes; Planctomycetacia; Planctomycetales; Planctomycetaceae; Planctomyces*	Uncultured bacterium clone CBM02G06 (EF395759)	98
S-40-28	0	1	5	8	0	0	0	1	*Fusobacteria; Fusobacteriales; Fusobacteriaceae; Propionigenium*	*Pseudomonas* sp. An30H-SC-S (AB267465)	99
B-09-329	0	0	0	0	0	0	1	0	*Actionbacteria; Actinomycetales; Micrococcaceae*	Uncultured bacterium clone nbt63g05 (EU536611)	99
U-09-12	0	0	0	0	1	2	0	0	*Actionbacteria; Actinomycetales; Actinomycetaceae; Actinomyces*	*Actinomyces massiliensis* strain 4401292 (EF558367)	99
S-09-27	1	0	1	0	0	0	0	0	*Actionbacteria; Actinomycetales; Corynebacteriaceae; Corynebacterium*	Uncultured bacterium clone 193b2 (EF459974)	98
S-09-24	2	0	0	0	0	0	0	0	*Actionbacteria; Actinomycetales; Corynebacteriaceae; Corynebacterium*	Uncultured bacterium clone ELB16-004 (DQ015796)	98
L-40-22	0	0	0	1	0	0	0	0	*Bacteroidetes; Bacteroides; Bacteroideles*	Uncltured *Bacteroidetes* bacterium clone SIMO-1973 (AY711339)	93
S-09-22	1	1	0	0	0	0	0	0	*Bacteroidetes; Flavobacteria; Flavobacteriales*	Uncultured bacterium clone S11-95 (EU287278)	99
L-09-16	0	0	1	0	0	0	0	0	*Bacteroidetes; Sphingobacteria; Sphinobacteriales; Flexibacteraceae*	Uncultured bacterium clone 916-U-34 (EU438323)	96
S-40-9	0	1	0	0	0	0	0	0	*Bacteroidetes*	Uncultured bacterium clone SHFH644 (FJ203571)	97
S-40-20	0	1	0	0	3	0	0	2	*Firmicutes; Lactobacillales; Streptococcaceae;Streptococcus*	Uncultured *Streptococcus* sp. clone JD112	99
S-09-41	1	0	0	0	0	0	0	0	*Firmicutes; Lactobacillales; Aerococcaceae*	Uncultured bacterium clone LK3 (EF186765)	94
B-09-219	0	0	0	0	0	0	1	0	*Firmicutes; Lactobacillales; Aerococcaceae*	*Abiotrophia elegans* (AF016390)	99
U-40-11	0	0	0	0	0	2	0	0	*Firmicutes; Bacillales; Staphylococcus*	*Staphylococcus* sp. 15-1-1 (EU870379)	100
U-09-131	0	0	0	0	2	1	0	0	*Firmicutes; Bacillales; Bacillaceae; Bacillus*	*Bacillus* sp. E53-10 (AY583458)	99
L-09-18	0	0	1	0	0	0	0	0	*Firmicutes*	Uncultured bacterium clone DMI (AY590186)	88
B-09-283	0	0	0	0	0	0	1	0	*Firmicutes; Clostridia; Clostridiales; Clostridiaceae; Clostridium*	Uncultured low G+C Gram-positive bacterium clone PICO pp37 Rainbow 146 (AJ969455)	97
L-40-6	0	0	0	1	0	0	0	0	*Firmicutes; Clostridia; Clostridiales; Clostridiaceae; Clostridium*	*Clostridium tyrobutyricum* (L08062)	96
S-09-7	1	0	0	0	2	0	0	0	*Firmicutes; Clostridia; Clostridiales;*	*Sporotalea propionica* strain TmPM3 (AM258974)	98
U-09-93	0	0	0	0	1	0	0	0	*Firmicutes; Clostridia; Clostridiales; Acidaminococcaceae*	Uncultured *Selenomonas* sp. clone 201B03 (AM420017)	98
U-40-17	0	0	0	0	0	2	0	0	*Firmicutes; Clostridia; Clostridiales; Acidaminococcaceae*	Uncultured bacterium clone VA25L-27 (DQ639089)	100

Supplementary figure legend

Fig. S1. DAPI-stained cells observed in the coelomic fluids. Scale bar, 5 μm.

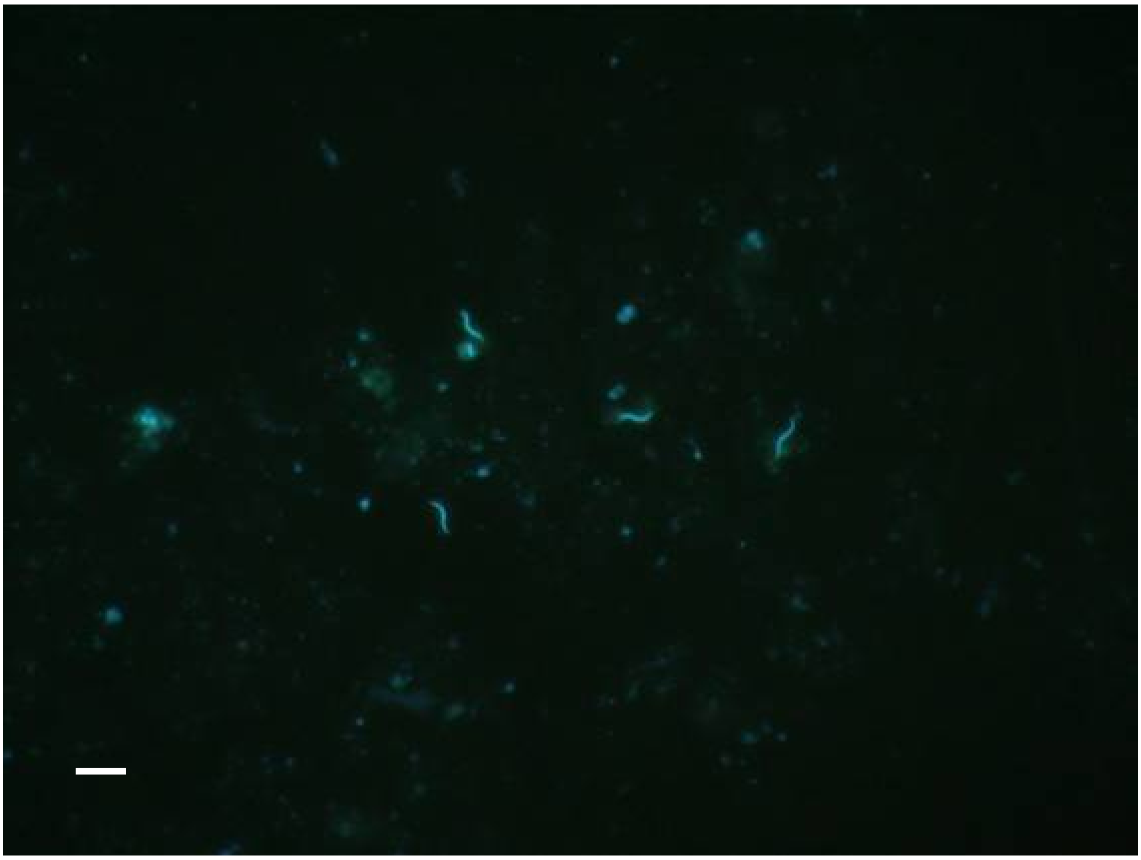

Fig. S1.
